# Smartphone Addiction and Associated Health Outcomes in Adult Populations: A Systematic Review

**DOI:** 10.3390/ijerph182212257

**Published:** 2021-11-22

**Authors:** Zubair Ahmed Ratan, Anne-Maree Parrish, Sojib Bin Zaman, Mohammad Saud Alotaibi, Hassan Hosseinzadeh

**Affiliations:** 1 School of Health and Society, Faculty of the Arts, Social Sciences and Humanities, University of Wollongong, Northfields Ave., Wollongong, NSW 2522, Australia; zar241@uowmail.edu.au (Z.A.R.); ma098@uowmail.edu.au (M.S.A.); 2 Department of Biomedical Engineering, Khulna University of Engineering and Technology, Khulna 9203, Bangladesh; 3 Department of Medicine, School of Clinical Sciences at Monash Health, Monash University, Melbourne, VIC 3800, Australia; sojib.zaman@monash.edu; 4 Department of Social Work, College of Social Sciences, Umm Al-Qura University, Mecca 24382, Saudi Arabia

**Keywords:** smartphone, addiction, health outcomes

## Abstract

Background: Smartphones play a critical role in increasing human–machine interactions, with many advantages. However, the growing popularity of smartphone use has led to smartphone overuse and addiction. This review aims to systematically investigate the impact of smartphone addiction on health outcomes. Methods: The Preferred Reporting Items for Systematic Reviews and Meta-Analyses (PRISMA) guidelines were used to carry out the systematic review. Five electronic databases including Medline, Web of Science, PsycINFO, PubMed, and Scopus were searched to identify eligible studies. Eligible studies were screened against predetermined inclusion criteria and data were extracted according to the review questions. This review is registered in PROSPERO (CRD42020181404). The quality of the articles was assessed using the National Institutes of Health (NIH) Quality Assessment Tool for Observational Cohort and Cross-Sectional Studies. Results: A total of 27 of 2550 articles met the inclusion criteria. All of the studies were cross-sectional and focused on physical, mental, and neurological health outcomes. The majority of the studies focused on mental health outcomes and consistent associations were observed between smartphone addiction and several mental health outcomes. Anxiety and depression were commonly found to mediate mental health problems. A wide range of physical health sequelae was also associated with smartphone addiction. Furthermore, there was an association between smartphone addiction and neurological disorders. Conclusions: Our findings suggest that there are consistent associations between smartphone addiction and physical and mental health, especially mental health. Social awareness campaigns about smartphone addiction and its impact on physical and mental health are needed. Further studies, especially randomized controlled trials, are warranted to validate the impacts of smartphone addiction.

## 1. Introduction

The 21st century is known as the age of information technology. Wireless communication and the internet are remarkable entities resulting in revolutionary changes in the field of communication [1]. In 2007, computer-based phones (smartphones) were introduced [2]. Since then, smartphones have become an indispensable part of daily life in all communities and countries. As such, smartphones have become one of the fastest-growing sectors in the technology industry [3]. Over the past decade, smartphone ownership and use have been exponentially increased globally. For instance, there were about 2.1 billion smartphone users in 2017 and the number was projected to exceed 2.8 billion by 2020 worldwide [4].

A number of novel problematic behaviors have emerged in the information technology era, such as gambling, internet gaming, and sexual behaviors, which may lead to compulsive engagement [5]. Extreme instances may lead to individuals feeling unable to control these behaviors without external influence, and these behaviors may be considered non-substance or behavioral addictions [6]. Internet addiction is one of the earliest examined forms of information technology addiction [7]. The relatively newer concept of “smartphone addiction” (SA) has also been studied based on previous internet addiction research [8]. Smartphones distinguish their use from traditional Internet use on computers or laptops because smartphones allow users to access the internet continuously regardless of time and space. Smartphone addiction is fueled by an Internet overuse problem or Internet addiction disorder [9]. The increased use of smartphones has resulted in most in people communicating daily online, as a result of interactive texts and social media, instead of face-to-face human contact. Smartphones fetch a limitless range of cognitive activities for users; smartphones forge opportunities for individuals to engage in a range of online activities such as participating in social network sites, playing video games, and “surfing the web” [10]. However, the smartphone poses a negative impact on our ability to think, remember, pay attention, and regulate emotion [11]. The increase in popularity and frequency of smartphone use has led to the emergence of clinical cases of people presenting with abuse symptoms [12].

The concept of addiction is not easy to define, and the usage of the term addiction has been considered controversial; however, central to its definition is the dependence on a substance or activity [13].

Smartphone addiction (SA) is generally conceptualized as a behavioral addiction including mood tolerance, salience, withdrawal, modification, conflict, and relapse [14]. Literature suggests that there are associations between SA and mental health [15], physical health [16], and neurological problems [17]. Furthermore, tolerance, salience, withdrawal, and cravings [8,18] have been associated with excessive smartphone use. However, the evidence is not conclusive [19]. Still, there is debate in the literature about the positive or negative relationship between the amount of screen time or smartphone use and health outcomes. Existing studies have provided useful data; however, it is difficult to draw consensus without a systematic review.

This systematic review is an attempt to collate empirical evidence about the health impacts of smartphone addiction among the adult population. This study aims to provide evidence to inform policy or recommendations to control and prevent smartphone addiction.

## 2. Methods

**Figure 1:**
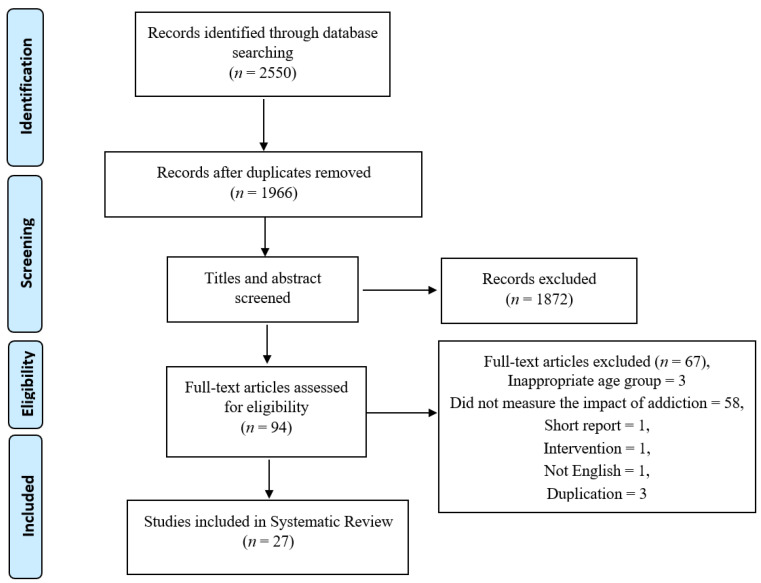
Preferred Reporting Item for Systematic Review (template taken from PRISMA flow diagram).

The protocol of this systematic review is registered in PROSPERO (CRD42020181404). It was carried out using the PRISMA (Preferred Reporting Items for Systematic Reviews and Meta-Analyses) guidelines (Figure 1). Literature searches were conducted in the five databases including Scopus, Medline, PubMed, Web of Science, and psycINFO databases. The search strategy for this review was initially developed by a series of consultations with the investigators and some preliminary searches (Z.A.R., A.M.P., S.B.Z., M.S.A., and H.H.). Expert librarians from the University of Wollongong were also consulted to refine and finalize the search strategy. All studies including controlled trials, case-control, cross-sectional, and cohort studies were included. Eligibility criteria included studies which explored smartphone exposure focusing on the adult population (aged over 18), published in the English language. This review excluded case reports, ideas, editorials, meta-analysis, review articles and opinions. Search terms included “smartphone”, “addiction”, “overuse”, “problematic use”, “excessive use”, and “adults”. Details of search strategies are provided in Supplementary Table S1. Since the smartphone gained popularity in 2011 (after the debut of the smartphone), the literature was searched from January 2011 until July 2021. The reference lists of the selected papers were also searched for any eligible papers however no papers were found.

Three authors (Z.A.R., S.B.Z., and M.S.A.) independently reviewed all the retrieved abstracts and selected eligible papers. Any disagreements were resolved by discussion with senior researchers (A.M.P. and H.H.). The quality of each included study was assessed by using the National Institutes of Health (NIH) Quality Assessment Tool for Observational Cohort and Cross-Sectional Studies and were given a rating of either “good”, “fair” or “poor” and the results of the quality assessment are presented in Supplementary Table S2. The NIH quality assessment is a valid and reliable tool for the assessment of the methodological quality of cross-sectional studies [20].

## 3. Results

### 3.1. Overall Search Findings

**Figure 2:**
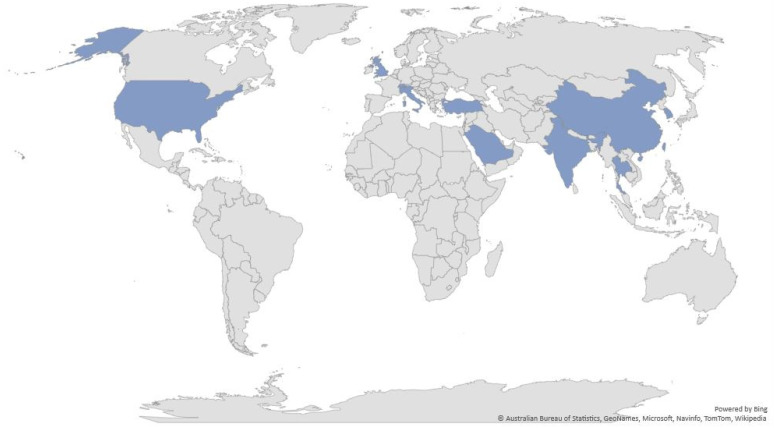
Global map indicating country of selected articles.

A total of 2550 potential studies were identified. After screening and removing duplicates, twenty-seven (27) studies were eligible for this review. A detailed study selection process based on the PRISMA flow chart is presented in Figure 1. Sample sizes ranged from 30 to 5372 adults (Table 1). Seven were conducted in South Korea [21,22,23,24,25,26,27], three in Saudi Arabia [28,29,30], four in China [31,32,33,34], four in Turkey [35,36,37,38], one in India [39] one in Taiwan [40], one in Switzerland [41], one in the USA [42], one in Italy [43], one in Thailand [44], and three were international studies [45,46,47] (Figure 2). Smartphone addiction was measured in the study sample using different scales, however, the Smartphone Addiction Scale, Short Version (SAS-SV; *n* = 8) was the most common measure (Table 1). Among the selected studies, nine studies were considered to be “good”, seventeen articles were considered to be “fair”, and the remaining one was considered “poor” (Table 2).

### 3.2. Main Findings

#### 3.2.1. Mental Health

As outlined in Table 2, mental health was associated with SA in fourteen studies [22,25,27,28,30,31,33,36,40,41,42,45,46,47]. Depression and anxiety were the most common mental health conditions associated with SA [22,25,28,30,31,33,36,41,45,47]. Several depression measures were used; however, the Beck Depression Inventory was the most common measure used [28,30,36,40]. Alhassan et al. (2018) revealed that less-educated people and young adult users of the smartphone were at high risk of depression. Another study [28] found that the groups who were classified as smartphone-addicted had an increased risk of depression (relative risk 1.337; *p* < 0.001) and anxiety (relative risk 1.402; *p* < 0.001) [28]. Miles Richardson et al. (2018) found that problematic smartphone use (PSU) was positively related to anxiety [46].

Social anxiety was also associated with SA [41]. For instance, a study conducted in China during COVID-19 reported that COVID-19 anxiety was associated with the severity of problematic smartphone use [33].

Interestingly, female participants were more susceptible to SA [36] and showed significantly higher dependence on smartphones than men [25]. Further, a study conducted among university students in Thailand demonstrated that not only were female students more likely to be smartphone addicted, but smartphone addiction among female participants was likely to be negatively associated with psychological well-being [44].

#### 3.2.2. Physical Health

##### Musculoskeletal Problems

The effect of SA on the musculoskeletal system was identified in four studies [24,26,34,43] (Table 2). Among those studies, two studies reported cervical problems [24,34], one study demonstrated nerve thickness [26], and one study showed psoriatic arthritis [43]. Lee et al. (2014) compared cervical spine repositioning errors in different smartphone addiction groups and revealed that there were significant differences between non-addicted, moderately addicted, and severely addicted groups; the severe smartphone addict group showed the largest changes in posture, the cervical repositioning errors of flexion (3.2 ± 0.8), extension (4.9 ± 1.1), right lateral flexion (3.9 ± 1.0), and left lateral flexion (4.1 ± 0.7). [24]. A study conducted among 2438 young patients suffering from chronic neck pain found that cervical disc degeneration was more likely to be associated with SA [34]. Another study conducted among university students revealed that excess smartphone use can cause nerve injury [26]. Megna et al. (2018) found that SA was linked to higher signs of inflammation in the musculoskeletal structures of hand joints.

##### Sleep Quality and Sedentary Lifestyle

Five studies showed an association between smartphone addiction and sleep quality [29,35,38,39,40]. The Pittsburgh Sleep Quality Index (PSQI) was used in all five studies (Table 1). A study conducted by Fahad et al. (2016) among 2367 university students reported 43% of the participants had decreased their sleeping hours due to SA, and 30% of the participants had an unhealthy lifestyle including weight gain, reduced exercise, and the consumption of more fast food when diagnosed with SA [29]. Another study conducted among migraine patients reported that SA can increase headache duration and decrease sleep quality [35].

##### Accidents

One study conducted by Hye-Jin Kim et al. (2017) revealed that SA is associated with different types of accidents, such as traffic accidents; falls/slips; bumps/collisions; being trapped in the subway, impalement, cuts, and exit wounds; and burns or electric shocks [21]. The study found that self-reported experience of accidents was significantly associated with SA [21].

##### Neurological Problems

Two studies reported the neurological effect of SA [23,32]; one study found alterations in white matter integrity [32] and another study reported smaller grey matter volume [23]. Hu et al. (2017) used a high-resolution magnetic resonance imaging technique to identify white matter integrity in young adults with SA and found that smartphone-addicted participants had significantly lower white matter integrity [32]. Lee et al. (2019) found that smartphone-addicted participants had significantly smaller grey matter volume (GMV) in the right lateral orbitofrontal cortex (OFC) [23].

## 4. Discussion

In recent years, several articles have examined the role of smartphone addiction and associated health outcomes among the adult population, however, substantial gaps still remain. To the best of our knowledge, no previous systematic review has been conducted to summarize these findings among this cohort. Our review is the first systematic review that utilizes empirical evidence from the last decades that demonstrates the relationship between smartphone addiction and health outcomes among adults. Interestingly, studies conducted in different parts of the world showed similar effects on health outcomes as a result of smartphone addiction. Hence, the consistency across the studies strengthens the study findings, emphasizing the association between SA and health outcomes.

Our findings suggest that depression and anxiety are significantly linked with smartphone addiction. One national USA survey found that 46% of smartphone owners believed they could not live without their phones [48]. Overuse patterns of smartphones involves a tendency to check notifications all the time, and such behavior patterns can induce “reassurance seeking” which broadly includes symptoms such as depression and anxiety [49]. This “reassurance seeking” pathway corresponds to those individuals whose smartphone use is driven by the necessity to maintain relationships and obtain reassurance from others. Bilieux and colleagues explained this reassurance-seeking behavior with the theoretical model of “problematic mobile phone use” [50]. In addition, this checking behavior is related to the next pathway, the “fear of missing out” (FOMO). One study found that FOMO mediated relations between both depression and anxiety severity with SA [51].

From our results, it is evident that musculoskeletal pain and insomnia are the two most common physical problems related to SA. Fingers, cervical, back, and shoulder problems are most commonly linked to excessive smartphone usage. Prolonged use of smartphones can cause defective postures such as forwarding head posture, which can produce injuries to the cervical spine and cause cervical pain [52]. Numerous studies found De Quervain tenosynovitis (characterized by pain in the wrist over the radio styloid process—the thumb side of wrist) was associated with different electronic devices like gaming controllers, tablets, and smartphones [53,54]. Texting and chatting through smartphones have been considered a risk factor for De Quervain tenosynovitis [55].

Poor sleep quality and difficulty in falling asleep or maintaining sleep has been identified as one of the negative consequences of SA, which is similar to our results [56,57]. Moreover, in line with our finding, another systematic review revealed that SA is related to poorer sleep quality [58]. One study found that 75% of the young adults (age < 30 years) take their phones to bed, which may increase the likelihood of poor sleep quality [59]. Smartphone addicts are unsuccessful at controlling their smartphone use, even in bed. Again, fear of missing out could be the reason of taking phones in the beds as they do not want to miss any notification [60,61]. In addition, blue light emitted by smartphones can have a negative effect on circadian rhythms, leading to negative sleep consequences, such as going to sleep later than intended and thus reducing overall sleep time [62].

The neurological effect of SA is not clear yet from this review. However, currently neuroimaging studies play an important role in understanding the complexity of addictive behavior [63], as they can assess any pathological change in the brain. Two studies in this review reported the negative changes in grey matter and white matter integrity in the brain with the assistance of neuroimaging (Table 2), which is similar to the neuropathy caused by substance abuse [64,65] and Internet addiction [66,67]. However, the modest sample size and the lack of a clinical evaluation are the potential limitations of these studies [23,32].

This review indicates that smartphone addiction shares similar features with substance abuse. A consistent relationship has been demonstrated between SA and physical and mental health symptoms, including depression, anxiety, musculoskeletal problems, and poor sleep. However, smartphones have become a part of daily life, facilitating work, education, or entertainment. Therefore, it is important not only to utilize the advantages of the smartphone but also to reduce the negative consequences. To address SA in a proper way, a validated definition and consistent diagnostic criteria of SA is required. The findings from this research suggest that healthcare providers and policymakers should recognize the problem and take necessary steps in raising community awareness about SA and its physical and mental impact.

## 5. Limitations

This systematic review has several limitations. First, all of the selected studies were cross-sectional (Table 1), therefore drawing conclusions about causal directions of associations is not possible. Secondly, all the papers were excluded if not in the English language; however, SA has received attention in Asian and European countries, and findings may have been published in other languages. This may lead to exclusion of studies conducted in diverse cultures and may bias the results of the review. Thirdly, most of the studies that were qualified to be included in this review were performed in developed countries, which may question the generalizability our findings to developing countries. Finally, most of the outcomes were reported over less than one year of follow-up. No standard scale and cut-off scores were used for the determination of smartphone addiction.

## 6. Conclusions

The current review describes the effect of smartphones on health outcomes in the adult population. Although the diagnostic criteria and effect of smartphone addiction are yet to be fully established, this review provides invaluable findings about the health impact of smartphone addiction and has significant implications for policy and decision makers. There is a need for more longitudinal studies to validate and strengthen this review’s findings. 

## Figures and Tables

**Table 1:** Smartphone addiction and associated health outcomes.

Authors, Country, Year	Sample Size	Type of Population	Age/Age Range	Gender	Type of Study	Outcome Measurement Tool	Pattern of Survey	Assessment Tool (SA)
Hye-Jin Kim [21,47], South Korea, 2017	608	University/college students	Control:23.01 ± 2.32, SA: 22.54 ± 2.05	Male = 183, Female = 425	Cross-sectional	Self-reported experience of accidents was assessed	Online questionnaire-based survey	SAPS
Yeon-Jin Kim [22], South Korea, 2015	4854	General	Age range 19–49	Male = 2573, Female = 2281	Cross-sectional	The Symptom Checklist-90-Revised-SCL-90-R	Online survey	K-scale
Deokjong Lee [23], South Korea, 2019	94	General	22.6 ± 2.4 (Age range 16–27)	Male = 61, Female = 27	Cross-sectional	Magnetic resonance imaging (MRI) scan	Online advertisements, MRI	SAPS
JeonHyeong Lee [24], South Korea, 2014	30	University students	N = 22.6 ± 1.3, Moderate Addiction Group (MAG) = 21.5 ± 1.9, Severe Addiction Group (SAG) = 22.4 ± 2.0	Male = 12, Female = 18	Cross-sectional	Motion meter (Performance Attainment Associates, West Germany)	Survey, the range of motion (ROM), a range of motion meter (Performance Attainment Associates, West Germany)	SAPS
Kyung Eun Lee [25], South Korea, 2016	1261	University/ college students	M 23.6 ± 2.7, F 21.5 ± 2.7	Male = 725, Femle = 511	Cross-sectional study	Zung’s Self-Rating Anxiety Scale	Face-to-face interview	Young’s Internet Addiction Test
Yeon-Seop Lee [26], South Korea, 2012	125	General	21.4 ± 2.0	Male = 32, Female = 93	Cross-sectional	Phalen’s tests, Reverse Phalen’s tests, Ultrasonography	Structured questionnaires	Structured questionnaires
Mi Jung Rho [27] South Korea, 2019	5372	General	26.43 ± 5.954 (Age range 19–39)	Male = 2443, Female = 2929	Cross-sectional	Brief Self-Control Scale (BSCS), Generalized Anxiety Disorder (GAD)-7, Patient Health Questionnaire-9 (PHQ-9), and Dickman Impulsivity Inventory-Short Version (DII).	Web survey	S-Scale
Aljohara A. Alhassan [28], Saudi Arabia, 2018	935	General public	31.7 ± 10.98 younger age group (18–35 years), middle-age group (36–54 years), and older age group (≥55 years)	Male = 316 (33.8%), Female = 619 (66.2%)	Cross-sectional	The Beck’s Depression Inventory second edition	Web-based	SAS-SV
Alosaimi, F. D. [29], Saudi Arabia, 2016	2367	University students	not mentioned	Male = 43.6%	Cross-sectional	Not mentioned	An electronic self-administered questionnaire	PUMP
Dalia El-Sayed [30], Saudi Arabia, 2020	1513	University students	M = 20.58 (1.71)	Male = 825 (54.5%) Female = 688 (45.5%)	Cross-sectional	Taylor Manifest Anxiety Scale and Beck Depression Inventory	Not reported	The Problematic Use of Mobile Phones (PUMP) scale
Jon D. Elhai [31], China, 2019	1034	Young adults	19.34 ± 1.61	Male = 359, Female = 675	Cross-sectional	Depression anxiety stress scale-21 (DASS-21), Fear of missing out (FOMO) scale	Web survey	SAS-SV
Yuanming Hu [32], China, 2017	49	Young adults	Control: 23.07 ± 2.01, SPD: 22.11 ± 1.78	Male = 26, Female = 23	Cross-sectional	Tract-based spatial statistics (TBSS) analysis	Survey questionnaire	MPATS
Jon D. Elhai [33], China, 2020	908	General	Age averaged 40.37 years (SD = 9.27)	Male = 156, Female = 752,	Cross-sectional	Depression anxiety stress scale-21 (DASS-21) Generalized anxiety disorder scale-7 (GAD-7) for COVID-19 anxiety	Web-based survey	Smartphone addiction scale-short version (SAS-SV)
Linbo Zhuang [34], China, 2021	2438	Young patients	Age, 18–44 years	Male = 1085, Female = 1353	Cross-sectional study	Magnetic Resonance Imaging (MRI) examination, Cervical Disc Degeneration Scale (CDDS)	Not reported	Smartphone Addiction Scale (SAS)
Yasemin P. Demir [35], Turkey, 2019	123	Patients who had Migraine	>18 years and <65 years	Male = 69, Female = 54	Cross-sectional comparative	Migraine disability assessment (MIDAS) questionnaire, The Visual Analogue Scale (VAS), Migraine Quality of Life Questionnaire) 24-h MQoLQ, Pittsburgh Sleep Quality Index (PSQI), Epworth Sleepiness Scale (ESS)	Written survey questionnaire	PUMP
Kadir Demirci [36], Turkey, 2015	319	University students	Mean age = 20.5 ± 2.45 years Smartphone non-user group 20.8 ± 2.11 Low smartphone use group 20.7 ± 2.74 High smartphone use group 20.2 ± 2.31	Male = 116, Female = 203	Cross-sectional	Pittsburgh Sleep Quality Index (PSQI), Beck Depression Inventory (BDI), Beck Anxiety Inventory (BAI)	Not reported	PUMP
Ayse Gokce [37], Turkey, 2021	319	University Students	18–33, 21.03 ± 2.05	Male = 104, Female = 215	Cross-sectional study	The Liebowitz Social Anxiety Scale (LSAS); Eating Attitudes Test (EAT).	Face-to-face survey	Problematic Mobile Phone Use Scale
Betul Ozcan [38], Turkey, 2021	1545		21.39 ± 2.21 years	Male = 43.2%, Female = 56.8%	Cross-sectional study	Pittsburgh Sleep Quality Index (PSQI)	Not reported	Smartphone Addiction Scale-Short Version (SAS-SV)
S HariPriya [39], India, 2019	113	College students	22.15 ± 1.69 (Age range 19–25)	Male = 63, Female = 50	Cross-sectional study	Pittsburgh Sleep Quality Index (PSQI), International Physical Activity Questionnaire-Short Form (IPAQSF)	Written survey questionnaire	Self-reported questionnaire
Hsien-Yuan Lane [40], Taiwan, 2021	422	University students	20.22 (SD = 2.34 years)	Male = 79, Female = 343	Cross-sectional study	Tri-Dimensional Personality Questionnaire (TPQ), Chinese Version of the Pittsburgh Sleep Quality Index (CPSQI), Beck Depression Inventory (BDI), Beck Anxiety Inventory (BAI)	Online	Chen’s Smartphone Addiction Inventory
Anna Maria [41] Switzerland, 2021	240	Young adults	18–35 years old, Mean age = 23.33,	Male = 120, Female = 120	Cross-sectional	12-item Social Anxiety Scale, a question on the daily duration of smartphone use, a single-item measure of dispositional truth	Online	Smartphone Addiction Scale Short Version
Jon D. Elhai [42], USA, 2018	300	College students	19.87 ± 3.79	Male = 24.3%, Female = 75.7%	Cross-sectional	Penn State Worry Questionnaire-Abbreviated Version (PSWQ-A), Dimensions of Anger Reactions-5 (DAR-5) Scale	Web survey	SAS-SV
Matteo Megna [43], Italy, 2018	52	Psoriatic patients	26.9 ± 7.8 (age range 18–35)	Male = 24, Female = 28	Cross-sectional	Nail Psoriasis Severity Index (NAPSI), Early psoriatic arthritis screening questionnaire (EARP), ultrasound score	Face-to-face interview	SAS-SV
Arunrat TangmunkongvorakulI [44], Thailand, 2019	800	University students	18–24 (Age range 18–24)	Male = 395, Female = 405	Cross-sectional	Flourishing Scale (FS)	Face-to-face	Young’s Internet Addiction Test
Zaheer Hussain [45], Global (majority in the UK, 86%), 2017	640	General	24.89 ± 8.54 (Age range 13–69)	Male = 214, Female = 420	Cross-sectional	Spielberger State-Trait Anxiety Inventory (STAI) Short-Form	Online survey	Independent questionnaire (Problematic smartphone use scale)
Miles Richardson [46], 2018, Global (majority UK, 82.8%)	244	General	29.72 ± 12.16	Male = 90, Female = 149	Cross-sectional	Spielberger State-Trait Anxiety Inventory (STAI), Nature Relatedness Scale	Web survey	PSUS
Asem A. Alageel [47], worldwide, 2021	506	Postgraduate students	Age 21 years and above (21–24 = 9.41%, 25–29 = 35.88% 30–39 = 44.51%, >=40 = 10.20%)	Male = 158 Female = 348	Cross-sectional	Patient Health Questionnaire (PHQ9) for depression, Athens Insomnia Scale (AIS), the Fagerström Test for Cigarette Dependence Questionnaire (FTCd),The adult ADHD Self-Report Scale (ASRS-v1.1)	Online	Smartphone Addiction Scale (SAS)

**Table 2:** Summary of outcomes.

Author and Reference	Outcomes	Specific Outcome	Quality
HYE-JIN KIM [21]	Smartphone addiction was significantly associated with total accidents, falling/slipping, and bumps/collisions	Accident	Fair
Yeon-Jin Kim [22]	SA had a stronger relationship with depression and anxiety, stronger than IA	Depression and anxiety	Fair
DEOKJONG LEE [23]	Small GMV in the lateral orbitofrontal cortex (OFC) was correlated with an increasing tendency to be immersed in smartphone use	Gray matter abnormalities	Fair
JeonHyeong Lee [24]	Significant differences in the cervical repositioning errors of flexion, extension, and right and left lateral flexion were found among the Normal Group, Moderate Addiction Group, and Severe Addiction Group	Musculoskeletal problems	Fair
Kyung Eun Lee [25]	For both men and women, increases in smartphone dependency were associated with increased anxiety scores	Anxiety	Fair
Yeon-Seop Lee [26]	Using smartphones continuously over long periods raises pressure on the median nerve and increases the probability of occurrence of CTS	Carpal tunnel syndrome	Poor
Mi Jung Rho [27]	Mental health problems were related to problematic smartphone use: (1) self-control (66%), (2) anxiety (25%), (3) depression (7%), and (4) dysfunctional impulsivities (3%)	Psychiatric symptoms	Fair
Aljohara A. Alhassan [28]	Significantly higher smartphone addiction scores were associated with younger aged users. Factors associated with higher depression scores were high school-educated users (β = −2.03, adj. *p* = 0.01) compared to the university educated group and users with higher smart phone addiction scores (β = 0.194, adj. *p* < 0.001).	Depression	Fair
Alosaimi, F. D. [29]	At least 43% had decreased sleeping hours and experienced a lack of energy the next day, 30% had an unhealthy lifestyle (ate more fast food, gained weight, and exercised less)	Risk of sedentary behavior	Fair
Dalia El-Sayed [30]	A significant positive correlation was found between PUMP score and depression and trait anxiety scores, duration of owning a smartphone, and average duration of each daily call.	Depression and trait anxiety	Good
Jon D. Elhai [31]	35.9% of our sample reported that they felt tired during day due to late-night smartphone use, 38.1% of them acknowledged that their sleep quality decreased, and 35.8% admitted that they slept less than four hours due to smartphone use more than once	Anxiety	Good
Yuanming Hu [32]	A primary understanding of white matter characteristics in SPD indicated that the structural deficits might link to behavioral impairments	Lower white matter integrity	Fair
Jon D. Elhai [33]	COVID-19 anxiety correlated with severity of PSU, depression, and anxiety 12% of participants were identified with at least moderate depression, and 24% with moderate anxiety	COVID-19 anxiety	Good
Linbo Zhuang [34]	Cervical disc degeneration may be associated with excessive smartphone use	cervical disc degeneration	Good
Yasemin P. Demir [35]	There was a negative correlation between MPPUS and PSQI (r = −0.367, *p* less than 0.05); a strong positive correlation between MPPUS and ESS (r = 0.675, *p* less than 0.05); and a negative correlation between MPPUS and 24-h MQoLQ (r = −0.508, *p* less than 0.05)	Increased headache duration, poor sleep quality	Fair
KADİR DEMİRCİ [36]	Smartphone Addiction Scale scores of females were significantly higher than those of males Depression, anxiety, and daytime dysfunction scores were higher in the high smartphone use group than in the low smartphone use group	Depression, anxiety, and daytime dysfunction	Fair
Ayse Gokce [37]	There is a mild, significant, positive correlation between the PU and LSAS scores of the students who participated in the study No significant relationship was found between the PU and EAT scores in the study group Problematic Mobile Phone Use Scale total scores showed a significant correlation with smoking	Increased smoking	Fair
Betul Ozcan [38]	Frequency of poor sleep quality was significantly higher in students with smartphone addiction compared to others	Poor sleep quality	Good
S HariPriya [39]	A moderately positive significant correlation between smartphone addiction and sleep quality was shown	Poor sleep quality, less physical activity	Good
Hsien-Yuan Lane [40]	With addiction to smartphones, higher risk of psychological distress and poor sleep quality was found, which is inconsistent with a previous report that more and more young adults report poor sleep quality in a higher percentage when they become addicted to smartphones	Psychological distress, poor sleep quality	Good
Anna Maria [41]	Social anxiety was significantly and positively related to PSU	Social anxiety	Fair
Jon D. Elhai [42]	Worry and anger may be helpful constructs in understanding the phenomenology of PSU, and psychological interventions for worry and anger may offset PSU	Worry and anger	Good
Matteo Megna [43]	Smartphone overuse was found to be linked with higher signs of inflammation	Psoriatic arthritis	Fair
Arunrat TangmunkongvorakulI [44]	Female students had scores for psychological well-being that were, on average, 1.24 points higher than the scores of male students (*p* < 0.001)	Psychological well-being	Fair
Zaheer Hussain [45]	The average time spent on a smartphone per day was 190.6 min (SD = 138.6) Problematic smartphone use was positively related to time spent on the smartphone and anxiety	Anxiety	Good
MILES RICHARDSON [46]	PSUS was not found to have diagnostic ability for high levels of anxiety	Connectedness with nature and anxiety	Fair
Asem A. Alageel [47]	65.9% of the participants who were identified as having high smartphone use had no depression, whereas 10.3% had severe depression, 16.1% had moderately severe depression, and 7.7% had moderate depression A significant correlation between the severity of insomnia and smartphone use 47.8% of the participants with high smartphone use had ADHD symptoms	Insomnia, depression, adult ADHD	Fair

## Data Availability

Not applicable.

## Supplementary Materials

The following are available online at https://www.mdpi.com/article/10.3390/ijerph182212257/s1, Table S1. Electronic search strategy.
