# Supplementary material for: Smartphone Addiction and Associated Health Outcomes in Adult Populations: A Systematic Review
Source: Int J Environ Res Public Health. 2021 Nov 22;18(22):12257. doi: 10.3390/ijerph182212257 (PMC8622754; doi:10.3390/ijerph182212257)
Supplement: Supplementary file 1 [file ijerph-18-12257-s001.zip › ijerph-1425730 table s1.pdf]

Table S1. Electronic search strategy

Database	Search Terms	Records Identified
**PubMed**	((smartphone) AND ((((addiction) OR (overuse)) OR (problematic use)) OR (excessive use))) AND (adults) Filters: Full text, Humans, Adult: 19+ years, English, from 2011/1/1 - 2021/7/31	**234**
**Scopus**	(TITLE-ABS-KEY (smartphone) AND TITLE-ABS-KEY (addiction) OR TITLE-ABS-KEY (overuse) OR TITLE-ABS-KEY (problematic AND use) OR TITLE-ABS-KEY (excessive AND use) AND TITLE-ABS-KEY (adults) AND NOT TITLE-ABS-KEY (children)) AND PUBYEAR > 2010 AND (LIMIT-TO (PUBSTAGE , "final")) AND (LIMIT-TO (OA , "all")) AND (LIMIT-TO (EXACTKEYWORD , "Human")) AND (LIMIT-TO (LANGUAGE , "English"))	**491**
**Medline**	TI Smartphone AND TI Addiction OR TI Overuse OR TI Problematic use OR TI Excessive use AND TI Adults<br><br>Limiters - Full Text; Date of Publication: 20110101-20210731; English Language; Human; Age Related: All Adult: 19+ years; Scholarly (Peer Reviewed)	**365**
**Web of Science**	**TITLE:** (Smartphone) *AND* **TITLE:** (Addiction) *OR* **TITLE:** (Overuse) *OR* **TITLE:** (Problematic use) *OR* **TITLE:** (Excessive use) *AND* **TITLE:** (Adults) *NOT* **TOPIC:** (Children)<br><br>Refined by: LANGUAGES: (ENGLISH) AND Open Access: (OPEN ACCESS) AND Open Access: (All Open Access) AND DOCUMENT TYPES: (ARTICLE) AND LANGUAGES: (ENGLISH) AND Open Access: (All Open Access) AND DOCUMENT TYPES: (ARTICLE)	**1257**

**Psychoinfo**	Smartphone AND Addiction OR Overuse OR Problematic use OR Excessive use AND Adults<br>Limiters - Full Text; Published Date: 20110101-20200431; Peer Reviewed; Open Access; Age Groups: Adulthood (18 yrs & older); Language: English	**203**
